# Impact of early postoperative factors on changes in skeletal muscle mass after esophagectomy in older patients with esophageal cancer

**DOI:** 10.1007/s41999-022-00735-0

**Published:** 2022-12-31

**Authors:** Tsuyoshi Harada, Noriatsu Tatematsu, Junya Ueno, Yu Koishihara, Nobuko Konishi, Takuya Fukushima, Hisashi Fujiwara, Takeo Fujita, Nanako Hijikata, Ayako Wada, Aiko Ishikawa, Tetsuya Tsuji

**Affiliations:** 1 Department of Rehabilitation Medicine, National Cancer Center Hospital East, Kashiwa, Chiba Japan; 2 Department of Rehabilitation Medicine, Keio University Graduate School, Shinjuku-Ku, Tokyo Japan; 3 Department of Integrated Health Sciences, Nagoya University, Nagoya, Aichi Japan; 4 Department of Musculoskeletal Oncology and Rehabilitation, National Cancer Center Hospital, Chuo-ku, Tokyo Japan; 5 Department of Esophageal Surgery, National Cancer Center Hospital East, Kashiwa, Chiba Japan; 6 Department of Rehabilitation Medicine, Keio University School of Medicine, 35 Shinanomachi, Shinjuku-ku, Tokyo 160-8582 Japan

**Keywords:** Esophageal cancer, Esophageal surgery, Geriatrics, Skeletal muscle mass

## Abstract

**Background:**

Loss of skeletal muscle mass, measured by the skeletal muscle mass index (SMI), after esophagectomy negatively impacts prognosis. However, the information to develop novel supportive care options for preventing loss of skeletal muscle mass is limited. The purpose of this retrospective cohort study was to investigate the impact of early postoperative factors on change in SMI 4 months after curative esophagectomy in older patients with esophageal cancer.

**Methods:**

This study included 113 subjects who underwent esophagectomy between 2015 and 2020. Preoperative and postoperative SMI (cm^2^/m^2^) were calculated from computed tomography images. The percentage change in SMI 4 months after surgery (SMI%) was calculated as follows: ([postoperative SMI – preoperative SMI] ÷ preoperative SMI) × 100. Potential factors affecting percentage change of SMI after surgery were analyzed by multiple regression.

**Results:**

The mean SMI% was – 5.6%. The percentage change (per 1%) in quadriceps muscle strength in the first month after surgery (standardized *β* = 0.190, *p* = 0.048) impacted the SMI%, which was independent of age, sex, preoperative SMI, comorbidity, pathological stage, and neoadjuvant chemotherapy.

**Conclusion:**

Quadriceps muscle weakness in the first month after esophagectomy impacted the SMI% in a dose-dependent relationship.

## Key summary points

**Aim:**

The aim of this retrospective cohort study was to investigate the impact of early postoperative factors on change in skeletal muscle mass 4 months after curative esophagectomy in older patients with esophageal cancer.

**Findings:**

The change (per 1%) in quadriceps muscle strength in the first month after surgery (standardized *β* = 0.190, *p* = 0.048) impacted the change (per 1%) in skeletal muscle mass 4 months after surgery, which was independent of age, sex, preoperative skeletal muscle mass, comorbidity, pathological stage, and neoadjuvant chemotherapy.

**Message:**

We believe that our findings will progress the development of novel comprehensive rehabilitation, including exercise and nutrition therapy after the perioperative phase in older patients with esophageal cancer.

## Introduction

The 5-year survival rate of patients who undergo curative esophagectomy for esophageal cancer (EC) is 30–83% [1], which is worse than for other cancers. In addition, the number of older patients with EC has been growing, and older patients have poorer prognoses than younger patients [2, 3]. There is a global need for research into potential interventions to improve the prognosis and health of older patients with cancer.

In older patients with cancer, skeletal muscle mass is an important prognostic factor [4, 5]. It was recently reported that in older patients with EC, a change in skeletal muscle mass 4 months after surgery is an important factor for overall survival independent of preoperative skeletal muscle mass [6, 7]. Additionally, the previous study also showed that the association between postoperative change in skeletal muscle mass (per 1%) and overall survival in older patients with EC had a strong dose-dependent relationship [7]. Although preoperative factors, including pathological tumor stage and preoperative skeletal muscle mass, impacted postoperative changes in skeletal muscle mass in previous studies [6, 7], to our knowledge, no studies have investigated the impact of early postoperative factors on postoperative changes in skeletal muscle mass. Changes in skeletal muscle mass are associated with reversible factors, such as physical function and nutrition, and irreversible factors, such as disease and aging, in older adults [4, 8, 9]. If early postoperative physical function and nutrition affect changes in skeletal muscle mass 4 months after surgery in older patients with EC, appropriate supportive care and rehabilitation may prevent critical loss of skeletal muscle mass.

The present study aimed to investigate the impacts of early postoperative factors on changes in skeletal muscle mass 4 months after curative esophagectomy in older patients with EC.

## Methods

### Design and participants

This study was a retrospective cohort study in patients aged 65 years or older who had undergone curative esophagectomy and physical function examination for EC at the National Cancer Center East Hospital in Japan between September 2015 and December 2020. Esophagectomy with three-field lymph node dissection was performed via open surgery or minimally invasive surgery. Enteral feeding was administered through a feeding tube placed in the jejunum in all patients. Seven days after surgery, all patients underwent a contrast study to identify any anastomotic leakage. If there was no leakage, oral fluid intake was started immediately. In patients with clinical stage II and III (Union for International Cancer Control tumor–node–metastasis [UICC-TNM] classification, 7th edition) EC, neoadjuvant chemotherapy (NAC) with cisplatin and fluorouracil or docetaxel, cisplatin, and fluorouracil was administered according to individual patient tolerance. Perioperative rehabilitation was performed on all patients. Preoperative rehabilitation was performed as home-based interventions consisting of respiratory training, resistance training, and aerobic exercise. Postoperative rehabilitation was performed from the first postoperative day at an intensive care unit up until discharge for 20–40 min per day, which included early mobilization, respiratory training, resistance training, and aerobic exercise.

The exclusion criteria were as follows: R1–2 curative esophagectomy; untreated or undertreated duplicate cancer at surgery; death or recurrence before the day of postoperative computed tomography (CT) at 4 months post-surgery; missing data. An opt‐out consent process was performed because of the retrospective nature of the study. This study was approved by the research ethics committee of the National Cancer Center (2019-075) in accordance with the Declaration of Helsinki. Informed consent was obtained through an opt‐out consent process.

### Assessment of patient characteristics

We obtained the following information from medical records: age; sex; NAC; histological tumor type; Charlson comorbidity index (CCI) [10]; pathological stage according to UICC-TNM classification, 7th edition; postoperative complications, including pneumonia and anastomotic leakage (≥ II according to Japan Clinical Oncology Group postoperative complications criteria, in line with the Clavien–Dindo classification [11]); and length of hospital stay (LOS).

### Assessment of physical function, nutrition, and inflammation

Isometric quadriceps muscle strength (QS; IsoForce GT-330, OG GIKEN, Japan) [12] and 4-m usual gait speed (UGS) [13] were collected as physical function indicators. For QS analysis, the side with a greater muscle strength before surgery was analyzed. Body mass index (BMI) and prognostic nutrition index (PNI) [14] were collected as nutritional indicators. C-reactive protein (CRP) [15] and neutrophil–lymphocyte ratio (NLR) [16] were collected as inflammation indicators. All factors were measured within 3 months before surgery and at first month after surgery. Percentage changes in QS, UGS, BMI, and PNI were calculated as follows: ([postoperative – preoperative] ÷ preoperative value) × 100. The postoperative change in CRP and NLR was measured as a bi-variable; postoperative CRP and NLR were defined by cutoff points of 0.5 mg/dL and 3.5, respectively [15, 16].

### Assessment of percentage change in SMI

The skeletal muscle mass index (SMI) [17] was calculated from CT images at the level of L3. CT was performed twice within 3 months, prior to and 4 ± 2 months after surgery. Regarding preoperative CT images, CT images after NAC were used if the patient was treated with NAC. The cross-sectional area (Hounsfield unit, –29 to 150) on CT images was measured in the skeletal muscle area using SliceOmatic (Imagelabo, Canada). The percentage change in SMI 4 months after surgery (SMI%) was calculated as follows: ([postoperative – preoperative] ÷ preoperative value) × 100.

### Statistics

Descriptive statistics are presented as number of patients, mean ± standard deviation, and median (1st–3rd quartile). SMI, QS, UGS, BMI, PNI, CRP, and NLR were compared before and after surgery by a paired *t* test or Wilcoxon signed-rank test to confirm postoperative changes. Variables without differences pre- and post-surgery were excluded from the analysis. The correlations of all factors were analyzed with the Spearman’s rank correlation coefficient. Associations between SMI% and postoperative factors were analyzed by simple linear regression. Multiple linear regression was performed using the forced entry method. Explanatory variables were potential factors with a *p* < 0.2 in the univariate analysis. Subsequently, age, sex, preoperative SMI [7], pT, pN [6], histological type [18], CCI [10], and NAC [19] were selected as potential preoperative confounding variables. The characteristics of the significant postoperative factors in the multiple linear regression were analyzed using a one-way analysis of variance or *χ*2 test. Statistical significance was considered as a two-tailed *p* value < 0.05. All analyses were performed with SPSS version 26 (IBM Corp., Japan) for Windows.

## Results

### Patient characteristics

**Fig. 1:**
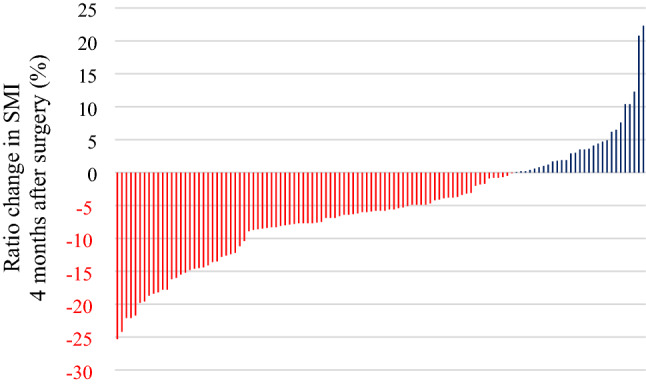
The individual percentage change in SMI 4 months after surgery. The red bars and black bars indicate that percentage changes in SMI were < 0% and ≥ 0%, respectively (*n* = 113)

Of an initial 146 patients, 33 patients were excluded for the following reasons: 4 patients, R1–2 esophagectomy; 2 patients, duplicate cancer; 10 patients, death or recurrence; 17 patients, missing data. The final analysis included 113 patients (Table 1). There were differences between preoperative and postoperative values for SMI, UGS, QS, BMI, and CRP (Table 2). The mean SMI% was – 5.6% ± 8.5%; there was heterogeneity in individual SMI% values (Table 2; Fig. 1).

**Table 1:** Characteristics of patients (*n* = 113)

Age, years	74.9 ± 4.7
Sex	
Male	92 (81)
Female	21 (19)
Histological type	
Adenocarcinoma	10 (9)
Squamous cell carcinoma	103 (91)
Tumor location	
Cervical	2 (2)
Thoracic	103 (91)
Abdominal	8 (7)
Pathological stage	
< III	84 (74)
III–IV	29 (26)
Pathological T stage	
< 3	73 (65)
3–4	40 (35)
Pathological N stage	
0	70 (62)
1–3	43 (38)
Preoperative adjuvant therapy	
Neoadjuvant chemotherapy	55 (49)
Surgery alone	58 (51)
Charlson comorbidity index	
< 2 score	88 (78)
2 or higher	25 (22)
Surgery	
Open surgery of chest or abdomen	17 (15)
Minimally invasive surgery	96 (85)
Postoperative complication	
Overall	43 (38)
Pneumonia	14 (12)
Leakage of anastomosis	11 (10)
Length of hospital stay, days	19.4 ± 7.7

All variables are expressed as *n* (%) unless otherwise stated. Postoperative overall complications are grade II or higher by Clavien–Dindo classification

*SD* standard deviation

**Table 2:** Measured variables before and after surgery (*n* = 113)

Variables	Before surgery	After surgery	Percentage change (%)	*p* value
SMI (cm^2^/m^2^)	40.9 ± 7.0	38.6 ± 6.9	– 5.6 ± 8.5	< 0.001^a^
QS (N)	400 ± 117	363 ± 121	– 8.5 ± 15.6	< 0.001^a^
UGS (sec/m)	1.17 ± 0.22	1.13 ± 0.23	– 2.6 ± 17.1	0.016^a^
BMI (kg/m^2^)	21.5 ± 2.9	19.8 ± 2.6	– 7.7 ± 3.9	< 0.001^a^
PNI (score)	40.5 ± 3.5	37.2 ± 3.4	– 7.6 ± 9.7	< 0.001^a^
CRP (mg/dL)	0.13 (0.06–0.28)	0.18 (0.09–0.73)	–	< 0.001^b^
NLR (ratio)	2.50 (1.80–3.60)	2.90 (1.85–3.90)	–	0.068^b^

Overall variables are expressed as the mean ± standard deviation or median (1st–3rd quartile)

*BMI* body mass index, *CRP* C-reactive protein, *NLR* neutrophil–lymphocyte ratio, *PNI* prognostic nutritional index, *QS* quadriceps muscle strength, *SMI* skeletal muscle mass index, *UGS* usual gait speed

^a^Paired *t* test

^b^Wilcoxon signed-rank test

### Impact of early postoperative factors on SMI

None of the variables showed high correlations (*r* > 0.7; Table 3). Multiple linear regression analysis showed that the percentage change in QS in the first month after surgery significantly impacted the SMI% independent of age, sex, preoperative SMI, pT, pN, histological type, CCI, NAC, and change in BMI (Table 4). The coefficient of determination (*R*^2^) of the model was 0.218.

**Table 3:** The correlation matrix of postoperative and preoperative factors (*n* = 113)

	QS%	UGS%	BMI%	PNI%	CRP	Age	Sex	SMI	pT	pN	Type	CCI	NAC
Postoperative factors													
QS%													
UGS%	0.23*												
BMI%	0.04	0.14											
PNI%	0.19*	0.13	– 0.03										
CRP	– 0.18	0.00	– 0.13	– 0.44*									
Preoperative factors													
Age	– 0.22*	– 0.12	– 0.06	– 0.16	– 0.01								
Sex	0.15	0.11	– 0.13	0.06	– 0.02	– 0.02							
SMI	0.21*	0.08	– 0.11	0.14	– 0.12	– 0.06	0.53*						
pT	– 0.05	– 0.04	0.09	0.04	0.09	0.22*	– 0.27	– 0.21*					
pN	0.14	0.06	0.01	0.16	– 0.11	– 0.03	0.14	0.07	0.18				
Type	0.13	0.04	0.21*	– 0.14	– 0.12	0.01	– 0.07	– 0.11	– 0.01	– 0.07			
CCI	0.05	0.04	0.11	– 0.05	0.09	– 0.09	– 0.07	0.03	0.11	– 0.02	0.02		
NAC	0.14	0.04	0.09	0.18	– 0.06	– 0.21*	– 0.15	– 0.30*	0.11	0.47*	0.07	– 0.08	

*BMI%* percentage change of body mass index, *CCI* Charlson comorbidity index, *CRP* C-reactive protein, *NAC* neoadjuvant chemotherapy, *PNI%* percentage change of prognostic nutritional index, *QS%* percentage change of quadriceps muscle strength, *SMI* skeletal muscle mass index, *Type* histological type, *UGS%* percentage change of usual gait speed

*p value < 0.05 (Spearman’s rank correlation coefficient)

**Table 4:** Associated factors with percentage change of SMI 4 months after surgery (*n* = 113)

Variables	Univariate analysis	*p* value	Multivariate analysis	|*β*|	*p* value
	*B* (95% CI)		*B* (95% CI)		
Postoperative factors					
Percentage change of QS (%), per 1%	0.109 (0.008 to 0.210)	0.035	0.104 (0.001 to 0.207)	0.190	0.048
Percentage change of UGS (%), per 1%	– 0.011 (– 0.104 to 0.083)	0.824			
Percentage change of BMI (%), per 1%	0.417 (0.011 to 0.822)	0.044	0.364 (– 0.032 to 0.759)	0.166	0.071
Percentage change of PNI (%), per 1%	0.084 (– 0.080 to 0.248)	0.312			
CRP 0.5 or more mg/dL (vs. < 0.5 mg/dL)	– 1.379 (– 4.804 to 2.046)	0.427			
Preoperative factors (confounding factors)					
Age (years), per 1 year	– 0.211 (– 0.560 to 0.117)	0.197	0.002 (– 0.348 to 0.343)	0.001	0.990
Male (vs. female)	– 2.794 (– 6.875 to 1.287)	0.178	– 1.620 (– 6.123 to 2.884)	0.074	0.477
Preoperative SMI (cm^2^/m^2^), per 1 cm^2^/m^2^	– 0.185 (– 0.411 to 0.041)	0.107	– 0.042 (– 0.306 to 0.222)	0.034	0.754
pT 3–4 (vs. < 3)	1.621 (– 1.712 to 4.954)	0.337	0.577 (– 2.826 to 3.980)	0.032	0.737
pN 1–3 (vs. 0)	– 0.562 (– 3.857 to 2.733)	0.736	– 1.222 (– 4.412 to 1.967)	0.070	0.449
Squamous cell carcinoma (vs. adenocarcinoma)	– 1.514 (– 7.141 to 4.114)	0.595	– 4.290 (– 9.681 to 1.101)	0.143	0.118
CCI score 2 or more (vs. < 2)	– 1.208 (– 5.057 to 2.642)	0.535	– 1.359 (– 5.047 to 2.329)	0.066	0.466
NAC (vs. surgery alone)	6.144 (3.427 to 9.362)	< 0.001	5.324 (1.920 to 8.728)	0.313	0.002

*β* standardized partial regression coefficient, *B* partial regression coefficient, *BMI* body mass index, *CCI* Charlson comorbidity index, *CI* confidence interval, *CRP* C-reactive protein, *NAC* neoadjuvant chemotherapy, *PNI* prognostic nutrition index, *QS* quadriceps muscle strength, *SMI* skeletal muscle mass index, *UGS* usual gait speed

### Association of significant postoperative factors with other factors

All patients were divided into two groups with a median value (– 9.0%) of the percentage change in QS as the cutoff point. The early postoperative major decline in QS group (percentage change, ≤ – 9.0%) had significantly lower preoperative SMI%, a greater decline in UGS and PNI 1 month after surgery, and longer LOS than the minor decline in QS group (Table 5).

**Table 5:** Characteristics of early postoperative major decline in QS

Variables	Early postoperative major decline in QS *n* = 57	Early postoperative minor decline in QS *n* = 56	*p* value
Basic characteristics			
Age, years	75.5 ± 4.6	74.3 ± 4.8	0.175^a^
Male	43 (75)	49 (88)	0.160^b^
pStage III–IV	12 (21)	17 (30)	0.359^b^
pT Stage 3–4	19 (33)	21 (38)	0.790^b^
pN Stage 1–3	18 (32)	25 (45)	0.216^b^
Squamous cell carcinoma	51 (90)	52 (93)	0.763^b^
CCI score 2 or more	11 (19)	14 (25)	0.615^b^
Neoadjuvant chemotherapy	27 (47)	31 (55)	0.508^b^
Preoperative SMI (cm^2^/m^2^)	39.4 ± 7.2	42.5 ± 6.6	0.018^a^
Preoperative physical function, nutrition, and inflammation			
QS, N	400 ± 117	400 ± 117	0.986^a^
UGS, sec/m	1.17 ± 0.23	1.17 ± 0.22	0.856^a^
BMI, kg/m^2^	21.4 ± 2.9	21.5 ± 3.8	0.866^a^
PNI, score	39.9 ± 3.7	41.1 ± 3.3	0.071^a^
CRP 0.5 or more mg/dL	12 (21)	10 (18)	0.848^b^
NLR 3.5 or more ratio	16 (28)	19 (34)	0.638^b^
Postoperative factors			
Percentage change of QS, %	– 20.2 ± 8.6	3.4 ± 11.6	< 0.001^a^
Percentage change of UGS, %	– 6.0 ± 16.9	3.4 ± 11.6	0.034^†^
Percentage change of BMI, %	– 7.9 ± 4.0	– 7.5 ± 3.8	0.598^a^
Percentage change of PNI, %	– 9.8 ± 9.4	– 5.4 ± 9.6	0.016^a^
CRP 0.5 or more mg/dL	22 (39)	14 (25)	0.177^b^
NLR 3.5 or more ratio	19 (33)	22 (39)	0.644^b^
Open surgery of chest or abdomen	7 (12)	10 (18)	0.571^b^
Presence of overall complications	37 (65)	29 (52)	0.221^b^
Length of hospital stay, days	21.2 ± 8.7	17.5 ± 6.0	0.010^a^

All values are *n* (%) unless otherwise stated

*CI* confidence interval, *β* standardized partial regression coefficient, *CCI* Charlson comorbidity index, *SMI* skeletal muscle mass index, *QS* quadriceps muscle strength, *UGS* usual gait speed, *BMI* body mass index, *PNI* prognostic nutritional index, *CRP* C-reactive protein, *NLR* neutrophil–lymphocyte ratio, Overall complication: ≥ grade II according to Clavien–Dindo classification

^a^One-way analysis of variance

^b^*χ*2 test

## Discussion

We investigated the impacts of early postoperative factors on changes in skeletal muscle mass after curative esophagectomy in older patients aged ≥ 65 years with EC. The mean SMI% 4 months after esophagectomy was – 5.6%. As shown in the multiple regression analysis, early postoperative change in QS in the first month after esophagectomy impacted the change in SMI 4 months after esophagectomy, which was independent of age, sex, baseline SMI, tumor stage and type, comorbidity, and NAC.

To our knowledge, the present study is the first study to investigate the impact of early postoperative factors, such as physical function, nutrition, and inflammation, on changes in skeletal muscle mass after esophagectomy in older patients. Skeletal muscle mass is affected by irreversible factors, such as aging [4], sex [4], tumor stage [6], disease [4], and chemotherapy [19], as well as reversible factors, such as physical function [9], nutrition [20], and inflammation [20]. We found that early postoperative changes in QS impacted changes in skeletal muscle mass 4 months post-esophagectomy in a dose-dependent manner in older patients, which was independent of irreversible factors.

The mechanisms behind the impact of early changes in muscle strength on changes in skeletal muscle mass could include potential mechanical stress on muscle fibers. It is well known that changes in the mechanical stress on muscle fibers can impact muscle strength via neurological and metabolic mechanisms [21, 22]. In addition, long-term changes caused by mechanical stress on muscle fibers could alter muscle mass due to an alteration in the protein synthesis and degradation balance, resulting in muscle atrophy or hypertrophy [23]. In a previous study, during a short-term bed rest period of 1–3 weeks, changes in muscle strength were reported to be faster and greater than changes in skeletal muscle mass [24, 25]. Furthermore, previous large longitudinal cohort studies suggested that changes in muscle strength preceded changes in skeletal muscle mass in community-dwelling older adults [9, 26]. In the present study, the early postoperative major decline in QS group had a greater decline in UGS and longer LOS than the minor decline in QS group. UGS and LOS are reportedly associated with physical activity [27, 28]. Hence, the early postoperative change in QS may have been influenced by postoperative physical activity during the hospital stay and after discharge, which could have led to subsequent changes in SMI.

In the present, the early postoperative massive decline in QS was characterized by lower preoperative SMI and a greater decline in PNI in the first month after surgery compared to the group of minor decline in QS. Older patients with preoperative vulnerabilities, such as low SMI, may have low resilience for muscle strength recovery [4]. Regarding the association between muscle strength and nutrition, malnutrition, such as vitamin E, carotenoids, and selenium deficiencies, was associated with lower muscle strength in a previous study [29]. Therefore, our findings, which indicate that early postoperative massive decline in QS is associated with preoperative SMI and change in PNI, are consistent with previous reports. In a recent meta-analysis, comprehensive rehabilitation, such as exercise therapy with protein supplementation, for older adults with risk of sarcopenia and frailty was reported to improve muscle strength and skeletal muscle mass [30]. In addition, the mechanism of the prognostic impact of postoperative loss of SMI was suggested to be associated with progression of frailty [7]. Considering our findings and previous studies, we hypothesize that in older patients with EC, continuous postoperative comprehensive rehabilitation, including exercise and nutrition therapy after perioperative rehabilitation, may prevent the loss of skeletal muscle mass and progression of frailty after esophagectomy by improving muscle strength.

There are several limitations to the present study. First, this study was a retrospective cohort study conducted at a single center. To confirm our results with validity and generalizability, prospective multicenter studies conducted with larger sample sizes are needed. Second, potential postoperative factors that had a strong impact may not have been included in the analysis. We were unable to assess physical activity, dietary intake, or cognitive and social function because of the retrospective nature of the study. Furthermore, although there was heterogeneity in the individual SMI% (Fig. 1), the coefficient of determination (*R*^2^) for the multiple regression analysis was 0.218; this indicated that the fit of the model was poor. Hence, interpretation of the results must be performed carefully and with consideration of the limitations.

In conclusion, in 113 patients aged 65 years or older with EC, the change in QS in the first month after esophagectomy impacted changes in SMI 4 months after surgery in a dose-dependent relationship, which was independent of irreversible factors such as age, sex, preoperative SMI, tumor stage, histological type, comorbidity, and NAC.

## Data Availability

Due to the nature of the retrospective study, participants of this study did not agree for their data to be shared publicly. Thus, data is not available publicly.
